# Ex Vivo Smooth Muscle Pharmacological Effects of a Novel Bradykinin-Related Peptide, and Its Analogue, from Chinese Large Odorous Frog, *Odorrana livida* Skin Secretions

**DOI:** 10.3390/toxins8100283

**Published:** 2016-09-27

**Authors:** Jie Xiang, Hui Wang, Chengbang Ma, Mei Zhou, Yuxin Wu, Lei Wang, Shaodong Guo, Tianbao Chen, Chris Shaw

**Affiliations:** 1 Natural Drug Discovery Group, School of Pharmacy, Queen’s University Belfast, Belfast BT9 7BL, Northern Ireland, UK; jxiang01@qub.ac.uk (J.X.); c.ma@qub.ac.uk (C.M.); m.zhou@qub.ac.uk (M.Z.); t.chen@qub.ac.uk (T.C.); chris.shaw@qub.ac.uk (C.S.); 2 School of Pharmaceutical Sciences, China Medical University, Shenyang 110001, China; wanghuiheidi@126.com; 3 Department of Nutrition and Food Science, College of Agriculture and Life Sciences, Texas A&M University, 123A Cater Mattil Hall, 2253 TAMU, College Station, TX 77843, USA; shaodong.guo@tamu.edu

**Keywords:** bradykinin related peptide (BRP), B2 receptor, rat tail artery, agonist and antagonist, smooth muscle

## Abstract

Bradykinin-related peptides (BRPs) are one of the most extensively studied frog secretions-derived peptide families identified from many amphibian species. The diverse primary structures of BRPs have been proven essential for providing valuable information in understanding basic mechanisms associated with drug modification. Here, we isolated, identified and characterized a dodeca-BRP (RAP-L1, T6-BK), with primary structure RAPLPPGFTPFR, from the skin secretions of Chinese large odorous frogs, *Odorrana livida*. This novel peptide exhibited a dose-dependent contractile property on rat bladder and rat ileum, and increased the contraction frequency on rat uterus ex vivo smooth muscle preparations; it also showed vasorelaxant activity on rat tail artery smooth muscle. In addition, the analogue RAP-L1, T6, L8-BK completely abolished these effects on selected rat smooth muscle tissues, whilst it showed inhibition effect on bradykinin-induced rat tail artery relaxation. By using canonical antagonist for bradykinin B1 or B2 type receptors, we found that RAP-L1, T6-BK -induced relaxation of the arterial smooth muscle was very likely to be modulated by B2 receptors. The analogue RAP-L1, T6, L8-BK further enhanced the bradykinin inhibitory activity only under the condition of co-administration with HOE140 on rat tail artery, suggesting a synergistic inhibition mechanism by which targeting B2 type receptors.

## 1. Introduction

Bradykinin (BK) is a nonapeptide hormone first discovered in 1949 by Rocha et al. upon the basis of its inherent capability to dilate vessels [1]. It has subsequently been reported that, in mammals, endogenous BK is generated via the effect of kallikrein on kininogen, which was derived mostly from, but not restricted to, liver tissues [2,3]. Since the first amphibian canonical BK was reported in the defensive skin secretions systems in 1965 from *Rana temporaria* [4], numerous bradykinin-related peptides (BRPs), with extraordinary diverse primary structure flanked by *C*/*N*-terminal extension, were isolated and identified from the Ascaphidae, Hylidae, Ranidae and Bombinatatoridae families. Of note is that mammals, reptiles, birds, and even fish were found to produce structurally discrete BRPs [5]. The research that focused on elucidating the hypervariable primary structures of BRPs across vertebrates was initially conducted by Conlon and his colleagues, and found that the diverse BRPs presented in unique vertebrate taxa all have identical counterparts in certain amphibian species [3,6]. Following on from this, the hypothesis was proposed that taxon-specific BRPs might be the most effective ligands for activating taxon-specific receptor mediated signal pathways, and for the improper BRP-ligands, they were believed to act as antagonists of the receptors to suppress the downstream signalling. DNA sequences that encode a wide range of BRPs have been cloned from frog skin secretions of different genus and species, many of which have been proved to have myotropic activities by using diverse mammalian smooth muscle preparations [5].

BK triggers its effect mainly through two types of receptors, B1 and B2, which all belong to the G protein-coupled receptors family. Compared with B2 receptors, which are generally expressed in multiple tissues, B1 receptors are only constitutively expressed in certain tissues, such as spinal cord, brain, and artery endothelial cells in the aorta and lung [7]. While upon inflammatory stimulus, B1 receptor expressions in a variety of tissues are upregulated in response to NF-κB (extensively studied transcription factors involved in immune response and infection) activation [8].

Stimulation or inhibition of B1 or B2 receptors is thought to be related with many pathophysiological disorders. Therefore, development of agonists or antagonists for either B1 or B2 receptors or even both would provide researchers significant implications no matter in the fields of pharmacology or clinical therapy.

Here, we reported a novel BRP, RAP-L1, T6-BK, which was isolated and identified from the skin secretion of the Chinese large odorous frog *Odorrana livida* by using 3′RACE and 5′RACE “shotgun” cloning technique. The primary sequence was further confirmed via the MS/MS fragmentation sequencing approach. The synthetic replicates of this novel peptide and its analogue RAP- L1, T6, L8-BK, were characterized by multiple organ-bath based ex vivo rat smooth muscle tissue probes. The wild-type RAP-L1, T6-BK was found to stimulate rat bladder, ileum, uterus contractile and tail artery relaxant responses. By contrast, the analogue RAP-L1, T6, L8-BK completely abrogated these functions, and it showed strong inhibition effect upon the BK-induced rat tail artery relaxation. Further pharmacological analysis revealed that BK B2 receptors are highly likely to be involved in the rat tail artery related effects caused by this novel BRP and its analogue.

## 2. Results

### 2.1. Molecular Cloning of cDNA Encoding the Biosynthetic Precursor of the Novel Bradykinin-Related Peptide

**Figure 1:**
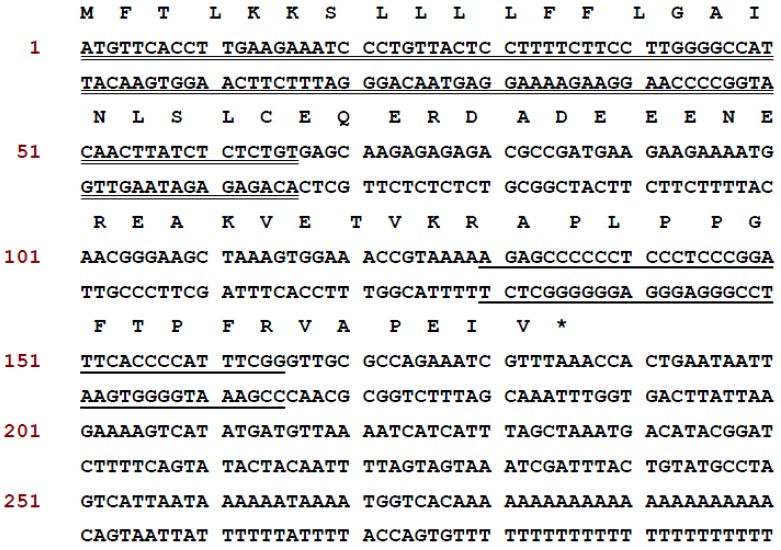
The nucleotide sequence and open-reading frame amino acid sequence of full length preprobradykinin-like peptide encoding cDNA from Chinese Large frog, *Odorrana livida*. The putative signal peptide is double-underlined. The mature peptide is single-underlined. The stop codon is indicated by an asterisk.

The preprobradykinin-like peptide encoding cDNA was consistently cloned from the *Odorrana livida* skin secretion-constructed cDNA library, the open-reading frame of this novel BRP precursor consists of 61 amino acids, and the architecture of translated open-reading frame can be divided into four domains. The 5′ *N*-terminus begins with a putative signal peptide with 22 amino acid residues followed by a 21 acidic residue-rich spacer, the putative 12-mer mature peptide is preceded by a propeptide convertase processing site -VK-, and it was followed by a *C*-terminal extension peptide (Figure 1).

### 2.2. Isolation and Structure Characterization of the Novel BRP

**Figure 2:**
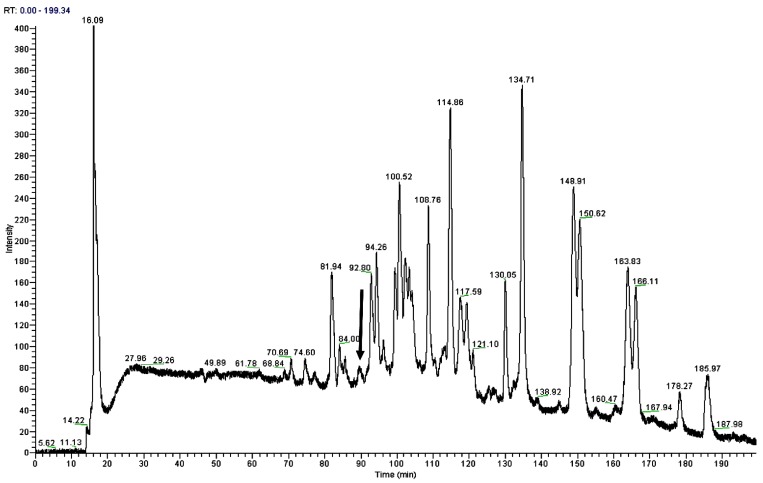
Region of reverse phase HPLC chromatogram of *Odorrana livida* skin secretion with arrow indicating the retention times (at 90 min) of the novel peptide RAP-L1, T6-BK. The detection wavelength was 214 nm with a flow rate of 1 mL/min in 240 min.

**Figure 3:**
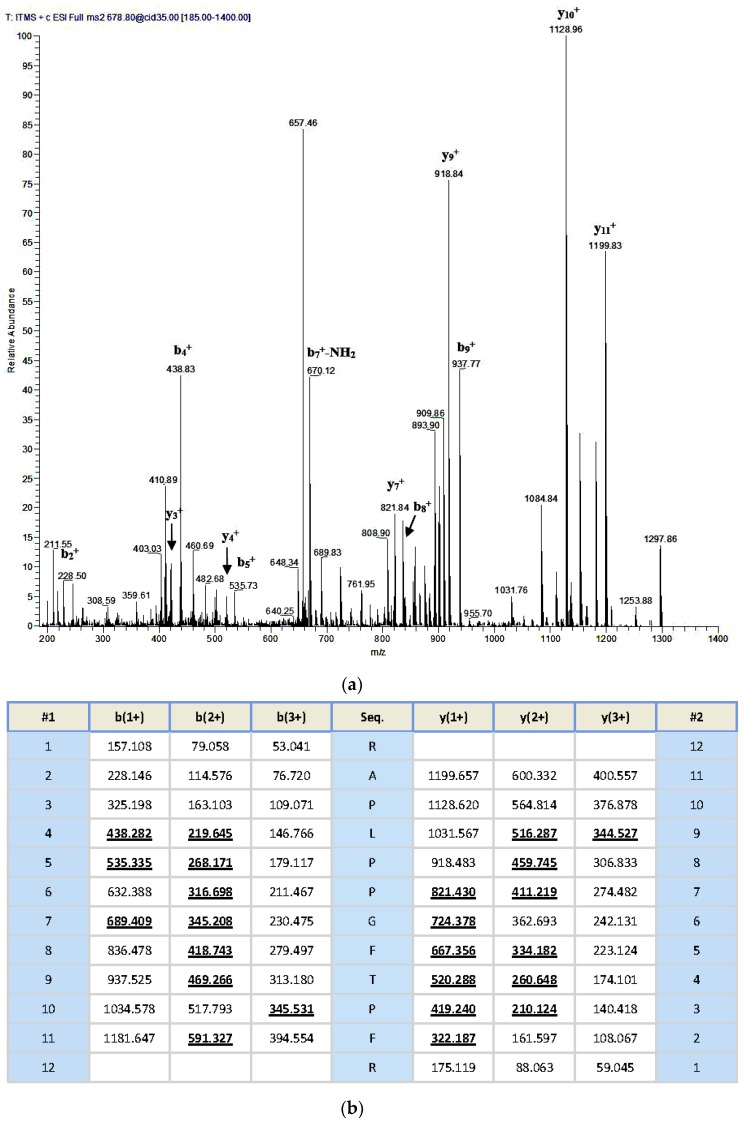
Thermoquest LCQ™ fragment scan spectrum derived from ions corresponding in molecular mass to RAP-L1, T6-BK (**a**) and electrospray ion-trap MS/MS fragmentation dataset (**b**) Expected singly- and doubly-charged b-ions and y-ions arising from MS/MS fragmentation were predicted using the MS Product programme available through Protein Prospector on-line. Truly observed ions are indicated in bold-typeface and underlined.

Reverse-phase HPLC chromatogram of the *Odorrana livida* skin secretion is shown in Figure 2. The fraction with the same mass of the peptide deduced from the molecular cloning (with calculated molecular mass 1355.59 Da) by using the specific BRP degenerate primer, which was described in Section 4.2, was identified by MS/MS fragmentation sequencing using the electrospray ion-trap mass spectrometer, the observed molecular mass was 1355.78 Da (Figure 3). Together with the result of the molecular cloning, the primary structure of the novel bradykinin-related peptide is unambiguously determined as RAPLPPGFTPFR, which is named as RAP-L1, T6-BK. Meanwhile, the analogue RAP-L1, T6, L8-BK (with calculated molecular mass 1320.59 Da) was confirmed by observation of its molecular mass of 1321.01 Da by using MALDI-TOF (Figure S2).

### 2.3. Bioinformatic Analysis of Novel BRP RAP-L1, T6-BK

**Figure 4:**
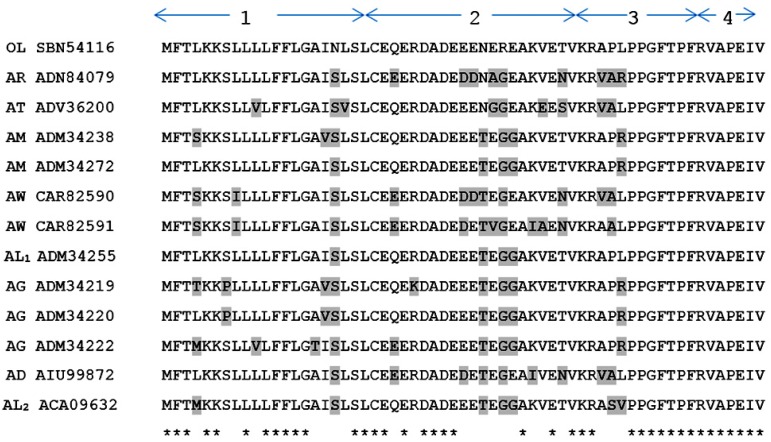
Alignment of cDNA-deduced RAP-L1, T6-BK precursor sequence and BRPs from *Amolops* species. Substitutions are highlighted in grey. Asterisks designate identical amino acid residues. Accession numbers are given in parentheses: (**1**) Putative *N*-terminal signal peptide domains; (**2**) acid spacer peptide domains; (**3**) mature peptide domains; and (**4**) *C*-terminal extension peptide domains. OL: *Odorrana livida*; AR: *Amolops ricketti*; AT: *Amolops torrentis*; AM: *Amolops mantzorum*; AW: *Amolops wuyiensis*; AL_1_: *Amolops lifanensis*; AG: *Amolops granulosus*; AD: *Amolops daiyunensis*; AL_2_: *Amolops loloensis*. * represents the highly conserved residues.

A BLAST search of this structure using the National Center for Biotechnological Information (NCBI) on line portal, revealed that the full length open reading frame of novel RAP-L1, T6-BK (OL SBN54116) peptide displayed relative high amino acid sequence identity (Query Cover: 100%; *E* value = 0.001; Identity: 64%–90%, including the Best Hits) with the BRPs precursor sequences from *Amolops* species. The highly-conserved domain includes the first residue Arginine of *N*-terminal extension and -PPGFTPFR- in the canonical BRP section (Figure 4). Detailed alignment was analysed using the AlignX program of the Vector NTI Bioinformatics Suite (Informax) (Figure 4).

### 2.4. Preliminary Study of the Inherent Cytotoxicity of Novel BRPs

**Figure 5:**
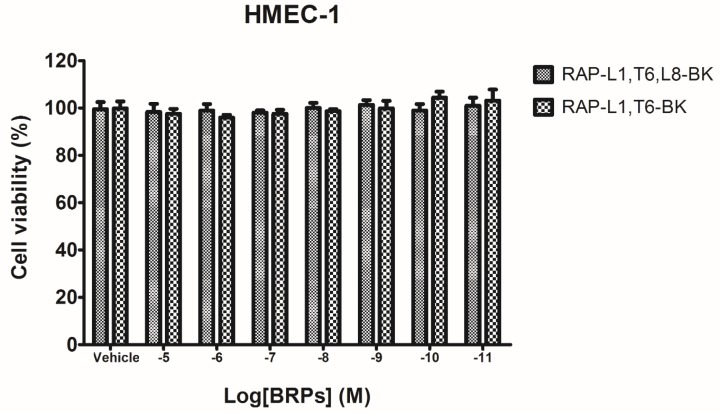
Assessment of cytotoxicity of BRPs. Dose–response curves of BRPs on human microvessel endothelial cells after a 24 h incubation. Each column represents the mean ± SEM of three replicates.

**Figure 6:**
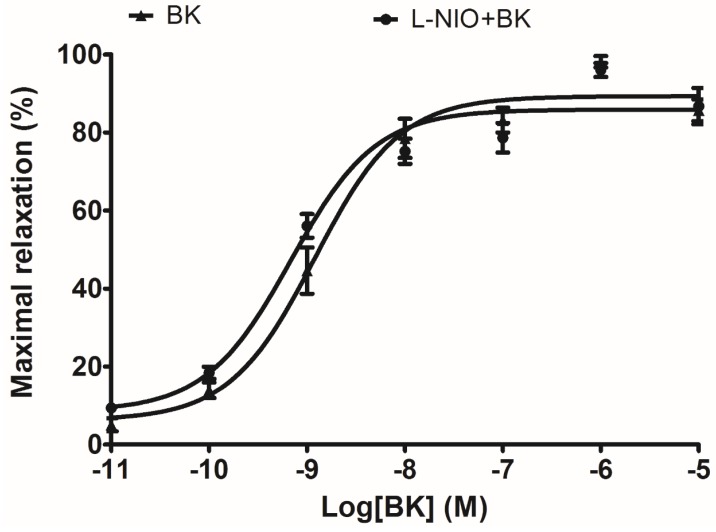
Assessment of the effects of eNOS on the smooth muscle effects of BK. Data represent the mean ± SEM of six replicates. There were no significant effects (*p* value 0.7562, two-way ANOVA) presented in the presence (●) or absence (▲) of the eNOS inhibitor, L-NIO, in each concentration compared to controls.

**Figure 7:**
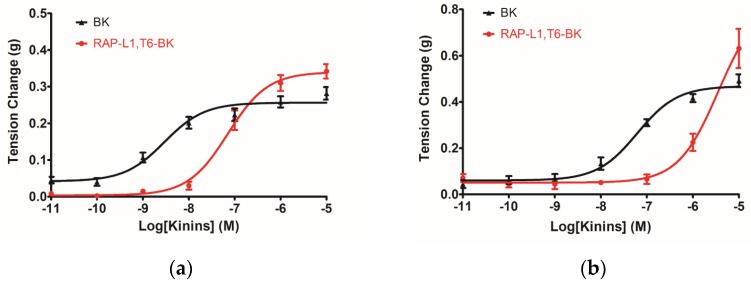

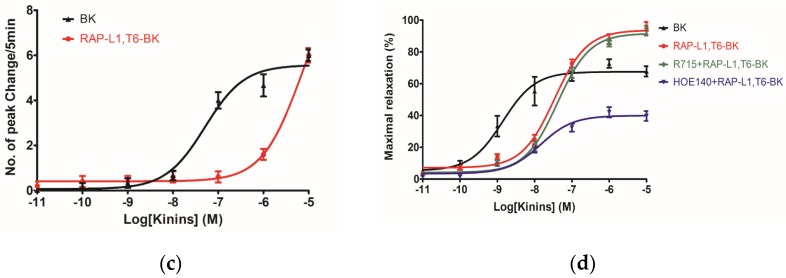
Dose–response curve of BK (black) and RAP-L1, T6-BK (red) induced contractile effects on rat bladder (**a**); and ileum (**b**); relaxant effects on tail artery preparations (**d**); and contraction frequency on uterus preparations (**c**). For rat tail artery treatment, RAP-L1, T6-BK dose–response curves in the presence of 10^−6^ M R715 (green) and 10^−6^ M HOE140 (violet) were presented accordingly.

**Figure 8:**
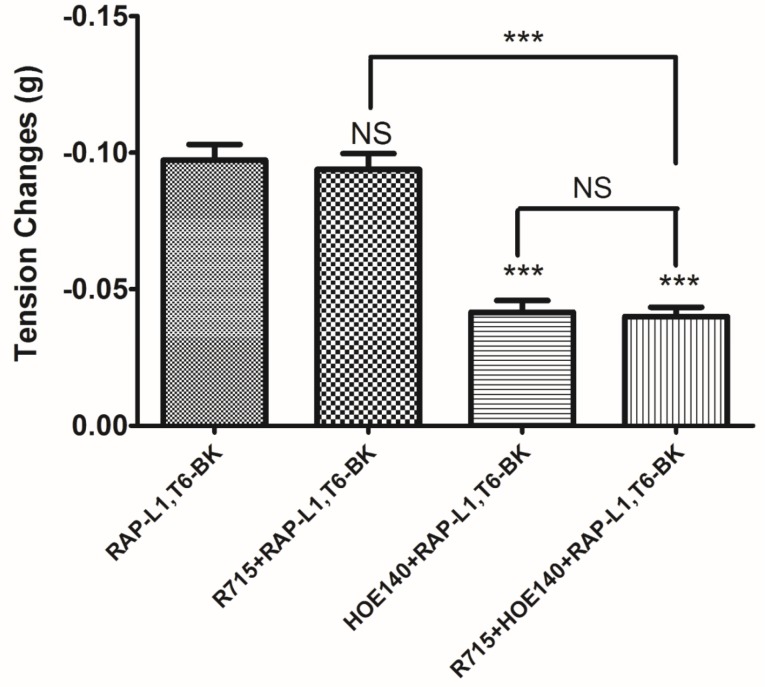
Rat tail artery smooth muscle tissues were pre-treated with R175 (10^−6^ M) or HOE140 (10^−6^ M) or their combination as indicated followed by administration of 10^−5^ M RAP-L1, T6-BK. (*** *p* < 0.001). All data points represent the mean ± SEM of five applications.

**Figure 9:**
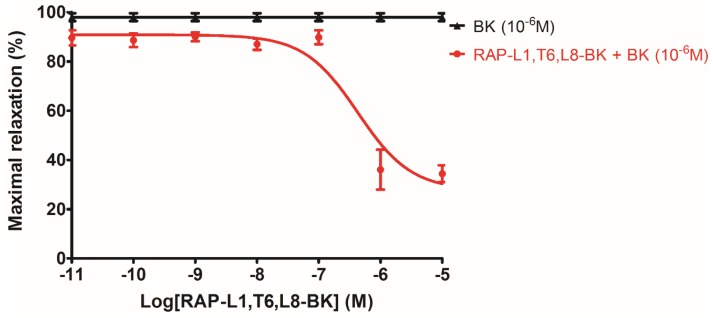
RAP-L1, T6, L8-BK dose-dependent inhibition response observed in the rat artery smooth muscle preparations followed by presence of maximal bradykinin effective concentration (10^−6^ M). Each point represents the mean and standard error of six replicates of three rats.

**Figure 10:**
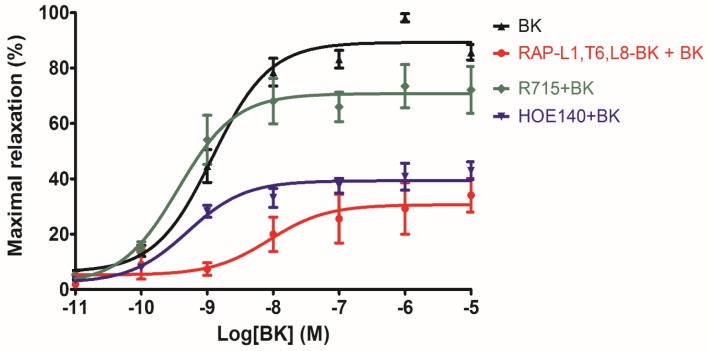
BK dose–response curves using rat arterial smooth muscle in the absence (black) and presence of RAP-L1, T6, L8-BK (red), HOE 140 (violet) or R715 (green) at a single dose of 10^−6^ M.

**Figure 11:**
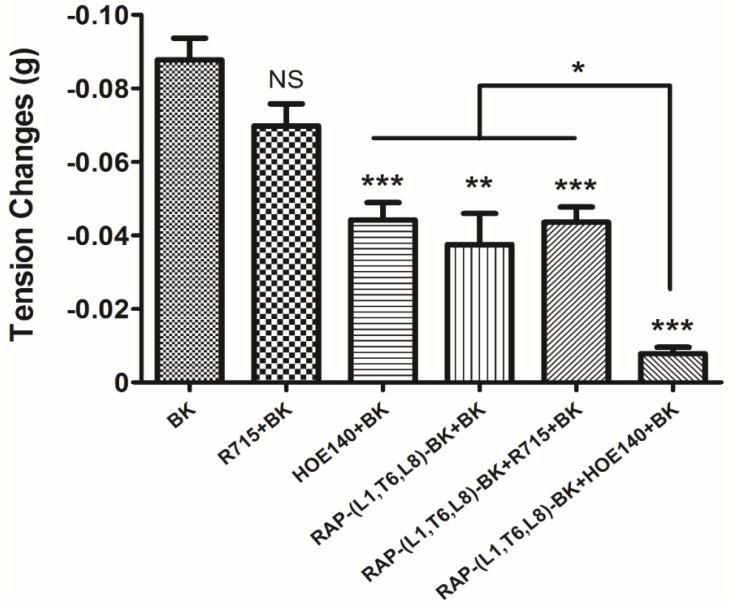
Rat tail artery smooth muscle tissues were treated by BK (10^−6^ M), R175 (10^−6^ M), HOE140 (10^−6^ M) single dose or their combination as indicated (* *p* < 0.05; ** *p* < 0.01; *** *p* < 0.001). All data represent the mean ± SEM of five applications.

The human microvessel endothelial cells (HMECs) were used to evaluate inherent cytotoxicity of two BRPs in this study. After incubation for 24 h, the viability of cells was evaluated using the MTT assay. The synthetic replicates of the BRPs exhibited no significant cytotoxic effects at the concentrations employed (10^−5^–10^−11^ M) in this assay (Figure 5), which is coincident with the molar concentrations used in pharmacological experiments (Figure 6, Figure 7, Figure 8, Figure 9, Figure 10 and Figure 11).

### 2.5. Smooth Muscle Pharmacology of Two Synthetic BRPs

Prior to the constructions of kinins dose–response curves, the eNOS inhibitor, L-NIO, was employed to exclude the endothelial-derived NO triggered artery relaxation. The results revealed that there is no significant difference (*p* value 0.7562, two-way ANOVA) on tissue responsiveness between BK-induced artery relaxation and L-NIO pre-administrated BK-induced artery relaxation, which confirmed that the NO was not likely to be involved in the effects of vasodilation under such rat tail artery smooth muscle preparation circumstances (Figure 6).

The novel BRP RAP-L1, T6-BK exhibited a dose-dependent activation of contraction in rat urinary bladder with EC_50_ = 3.54 ± 0.83 µM compared with BK (EC_50_ = 61.78 ± 0.93 nM) (Figure 7a). For rat ileum, the dose-dependent contractile effects of RAP-L1, T6-BK and BK were EC_50_ = 70.95 ± 0.98 nM and EC_50_ = 2.95 ± 0.12 nM, respectively (Figure 7b). On rat uterus smooth muscle preparations, RAP-L1, T6-BK significantly increased the number of contractions during 5 min intervals when the peptide concentration raised to 10^−6^ M and 10^−5^ M, with EC_50_ = 6.82 ± 0.75 µM versus BK EC_50_ = 51.02 ± 1.05 nM (Figure 7c). For rat tail artery, BK exerted more potency in relaxation effect with IC_50_ = 1.408 ± 0.15 nM versus RAP-L1, T6-BK with IC_50_ = 34.04 ± 2.35 nM (Figure 7d).

To further examine the preliminary vasodilation mechanism of RAP-L1, T6-BK, specific BK subtype receptor antagonists (R715 for typical BK B1 receptor antagonist; HOE140 BK B2 receptor antagonist) were pre-treated alone or in combination with RAP-L1, T6-BK in rat tail artery smooth muscle preparations. BK B2 receptor antagonist, HOE-140, significantly inhibited the RAP-L1, T6-BK induced relaxation of rat tail artery (*p* = 0.0002, *n* = 5), whereas specific BK B1 receptor antagonist R715 did not show any attenuation effect on RAP-L1, T6-BK induced rat tail artery relaxation (*p* = 0.1222, *n* = 5). In addition, the combined pre-treatment of HOE 140 and R715 exhibited no significant difference with the singular application of HOE 140 (*p* = 0.7931, *n* = 5) (Figure 8). These data indicated, as expected, that the RAP-L1, T6-BK -induced relaxation of the rat tail arterial smooth muscle was most likely to be mediated by BK B2 receptors.

The analogue RAP-L1, T6, L8-BK showed no effect on rat tail artery smooth muscle preparations from 1 × 10^−11^ to 1 × 10^−5^ M (Figure S1). However, when rat tail artery was pre-treated with RAP-L1, T6, L8-BK dose range from 1 × 10^−11^ M to 1 × 10^−6^ M followed by administration of BK at maximal effective concentration 10^−6^ M as described previously [9], maximum inhibition of BK-induced relaxation was observed at 10^−6^ M and 10^−5^ M. Hence, we selected 10^−6^ M as the most effective inhibitory concentration on rat tail artery smooth muscles (Figure 9). In the second series of experiments, when the rat tail artery smooth muscle preparations were treated with 10^−6^ M RAP-L1, T6, L8-BK followed by the application of BK concentration range from 10^−11^ M to 10^−5^ M, the potency of BK-induced maximum relaxation of rat tail artery smooth muscle at 10^−9^ M to 10^−5^ M was dramatically decreased (nearly 40%–60%), although the IC_50_ values were 1.20 ± 0.08 nM vs. 0.50 ± 0.56 nM. Similar to RAP-L1, T6, L8-BK, HOE140 pre-treatment largely antagonize BK-induced relaxation, whereas this effect was not detected under R715 pre-treatment (Figure 10).

To further assess the receptors that might be involved in the antagonism, co-administration of HOE140 or R715 with 10^−6^ M RAP-L1, T6, L8-BK was employed prior to induction of rat tail artery relaxation by 10^−6^ M BK. Consistent with the previous data, R715 had a trend to attenuate BK’s effect, but no statistic difference was determined (*p* = 0.0773, *n* = 5), RAP-L1, T6, L8-BK and HOE140 significantly attenuated rat tail artery dilation induced by BK (*p* = 0.0028 and 0.0006, *n* = 5, respectively). Co-administration of R715 with RAP-L1, T6, L8-BK did not further enhance the BK attenuation effect, while co-administration of HOE140 with RAP-L1, T6, L8-BK exhibited a synergetic inhibition activity to almost block BK-induced rat tail artery relaxation (*p* < 0.0001, *n* = 5) (Figure 11).

## 3. Discussion

Frog skin secretions contain large number of bioactive components, including antimicrobial, protease inhibitory, and pharmacological relevant peptides. As we stated in the introduction, discrete BRP may have unique ligand-receptor specificity and potency, which is vital to provide valuable structure-interaction relationship information for agonistic and antagonistic research. Therefore, we decided to focus on “shotgun cloning” novel BRP by using BK-derived degenerate primer in this project. In addition, BRPs from amphibian secretions and venoms are considered to be homologous counterparts of endogenous mammalian polypeptide hormones. Therefore, study on their physiological and pathological relevance is essential for us to understand metabolic homeostasis in vivo, which could provide us novel insight into cardiac, vascular, renal and inflammatory associated disorders [10,11,12,13].

In this study, we describe the structural and functional characterizations of a novel BRP first identified in the skin secretions of Chinese Large frog, *Odorrana livida*, through “shotgun” cloning of a biosynthetic precursor-encoding cDNA from a lyophilised skin secretion-derived cDNA library (Figure 1). Both the open-reading frame nucleotide sequences and amino acid sequences encoding the precursors of BRPs from *Amolops* and *Odorrana* (Figure 4) elicited unparalleled similarity (identity: 64%–90%), which suggests a possible evolutionary connection between these two genera of frogs [14]. The mature novel BRP, RAP-L1, T6-BK, has a three residue *N*-terminal extension RAP, and two site-substitutions L1, T6-BK compared with canonical BK. In addition, the T6-BK was found among several species of reptiles such as crocodilians, chelonians, and varanid lizards which contain whole components of the kallikrein-kinin system in mammals [6]. Threonine 6 is one of the most common site-substitutions of amphibian skin secretion-derived BRPs, which reflects their evolutional adaptations to particular predators [15]. It is believed that the replacement of Threonine at position 6 of BK is associated with enhanced effect of contracting rat ileum with equivalent potent to relax rat tail artery compared with BK [16,17]. Meanwhile, *N*-terminal or *C*-terminal extended BRPs are commonly identified from amphibian secretions and venoms. This study reported the -RAP- extension at N-terminal end BRP from *Odorrana livida*. The BRPs Amolopkinin-W1/W2 isolated from *Amolops wuyiensis* exhibited high degree of sequence similarity with RAP-L1, T6-BK, with the *N*-terminal extension motif structure -RAA- and -RVA-, respectively. Both the Amolopkinin-W1 and Amolopkinin-W2 exhibited inhibitory effect of BK-induced intestinal smooth muscle contraction, while no direct effects on bladder and uterine smooth muscle preparations were detected [14]. Despite the fact that the precise functions of diverse *N*- or *C*-terminal extension of BRPs still remains elusive, they are predicted to be involved in prolonging the interaction time of these peptides as ligands with target receptors, which could be adopted as a defensive strategy by host to survival [18,19].

In terms of artery relaxation, considering the fact that a lot of vasodilators were involved in the release of nitric oxide (NO) through a common downstream signalling, and NO is widely accepted as the final substance that induces smooth muscle relaxation and inflammatory status [20]. The eNOS (NO releaser) inhibitor, L-NIO, was pre-treated in arterial smooth muscle to test whether the well-established vasodilation via BK induction was influenced. Since the result did not show any significant difference, it implied that NO is not possible to be associated with this ex vivo rat tail artery smooth muscle model.

The myotropic potency of this novel identified RAP-L1, T6-BK peptide is relatively weak compared to BK, not only in contraction of rat bladder, ileum and uterus, but also in dilating vessels. Whilst, the novel peptide seemed to exhibited higher efficacy for the receptors compared to BK, which could be deduced from the Figure 7d that at the concentration of 10^−7^ M, RAP-L1, T6-BK have similar vasodilate capacity with BK, and when the concentration raised to 10^−6^ M and 10^−5^ M, RAP-L1, T6-BK dramatically enhanced the vasodilation on rat tail artery smooth muscle compared to BK, which indicated that the threshold concentration for RAP-L1, T6-BK and BK to induce a significant rat tail artery relax is differentiated, BK is more potency at lower concentration (10^−9^ M–10^−7^ M), but RAP-L1, T6-BK is more effective at higher concentration (10^−6^ M–10^−5^ M). In consideration of this, the primary structure similarity between them proposed us the hypothesis that RAP-L1, T6-BK mediates these effects through the cognate G protein-coupled receptors, and thus, we examined the arterial smooth muscle relaxing property modulated by RAP-L1, T6-BK in the presence of HOE140, a well-established BK B2 antagonist and R715, a selective BK B1 antagonist. Surprisingly, we observed a significant attenuation (nearly 60%) in HOE140 treatment condition, while R715 barely antagonize RAP-L1, T6-BK, which suggests a BK B2 receptors-related mechanism. Further experiments are still needed to address this issue thoroughly. Additionally, stimulation of neurokinin receptors in rat bladder, muscarinic receptors in ileal and endothelin receptors in uterine smooth muscle, which leads to intracellular calcium concentration, are reported to be involved in the contractions of these tissues [21,22,23,24,25], the relationship between RAP-L1, T6-BK and these receptors are worthy to be investigated in the future.

The analogue peptide RAP-L1, T6, L8-BK completely abrogated those bioactivities. Of interest is that only the leucine residue substitution at position 8 of BK would cause such an astonishing function loss. Leucine 1 is a common substitution occurred in BRPs, compared with valine substation at position 1, it normally exhibits moderate agonistic effect of BK [26]. As the fact that Leu8-BK was reported as a BK receptor antagonist [27], we performed further experiments to examine whether RAP-L1, T6-BK and its analogue RAP-L1, T6, L8-BK could inhibit BK-induced relax on rat tail artery smooth muscle, the results revealed that, as expected, RAP-L1, T6-BK did not show BK inhibition property (Figure S1), but RAP-L1, T6, L8-BK significantly reduced BK relaxation activity on rat tail artery nearly 50% in the concentration range 10^−8^ M–10^−5^ M. These data suggested that although this novel BRP has a three residues -RAP- extension and leucine 1, threonine 6 substitutions, the leucine 8 still plays the essential and dominant role in reversing the BK’s effect. To investigate the precise manner how RAP-L1, T6, L8-BK exert BK antagonistic effect, again, we pre-treated rat tail artery smooth muscle with specific BK B1 receptors and/or B2 receptors inhibitors. When the tissues pre-treated with R157, BK-induced artery relaxation has a trend to be attenuated, but no statistical differences were observed. In contrast, when the tissues were pre-treated with HOE140, BK-induced artery relaxation was largely impaired. This probably due to that B1 receptors are rare expressed in rat tail artery or B2 receptor-mediated signalling is mainly responsible for BK-induced relaxation in rat tail artery, further examination by pre-treating the tissues with RAP-L1, T6, L8-BK along or together with R157 showed similar BK inhibition potency, which implies that the peptide might exert the effect in a B2 receptors-dependent manner. While RAP-L1, T6, L8-BK pre-treatment along with HOE140 almost abolished BK-induced relaxation suggested a novel synergistic inhibition mechanism via the BK B2 receptors might be involved compared with HOE140. In terms of this additive effect, others receptors, rather than BK receptors, activated by RAP-L1, T6, L8-BK is also a feasible option. Therefore, the primary structure initiated ligands-receptor interaction study of BRPs could provide researchers valuable information for developing novel molecular therapeutics.

Taken together, due to the inherent characteristics of the physiopathological significance of diverse BRPs for their potential to treat multiple vascular, cardiac diseases, they have been focused on for many years. In this project, we reported a novel BRP and its analogue isolated from Chinese large frog *Odorrana livida*, which presented distinct bio-functionalities, have unique structure/activity relationship, and continuously provides virtues for drug design.

## 4. Materials and Methods

### 4.1. Specimen Preparation and Secretion Harvesting

Adult Chinese Large Odorous frogs, *Odorrana livida* (*n* = 3, the length from snout to vent was 8–10 cm) were obtained from the mountain areas of Fujian province, People’s Republic of China. The gentle electrical stimulation (5 V, 50 Hz, 4 ms plus width) (C.F. Palmer, London, UK) was performed to induce the secretion from the glands of the frog dorsal surface. After secretion harvesting, the frogs were released. This method was non-lethal to frogs. The distilled deionized water was used to rinse the frog skin surface for secretions collection. The secretions were then subjected to snap frozen with liquid nitrogen, lyophilized and stored at −20 °C before use. Sampling of skin secretion was performed by Mei Zhou under UK Animal (Scientific Procedures) Act 1986, project license PPL 2694, issued by the Department of Health, Social Services and Public Safety, Northern Ireland. Procedures had been vetted by the IACUC of Queen’s University Belfast, and approved on 1 March 2011.

### 4.2. “Shotgun” Cloning of a Novel BRP Encoded cDNA Library

Five milligrams of lyophilized *Odorrana livida* skin secretion was dissolved in lysis/Binding Buffer. The extraction of polyadenylated mRNA was performed by using Dynabeads^®^ mRNA DIRECT™ Kit (Dynal Biotech, Liverpool, UK). The cDNA library was obtained in reverse transcription reaction. Both 3′-RACE and 5′-RACE PCRs were employed to obtain the novel full-length prepro-BRP nucleic acid sequence by using SMART-RACE kit (Clontech, Oxford, UK). Briefly, the 3′-RACE PCR reaction employed a specific sense primer (5′-CCRVCNGGGTTYASSCCWTTY-3′) (R = A/G; V = A/C/G; N = A/C/T/G; Y = C/T; S = C/G; W = A/T) that was complementary to the nucleic acid sequence encoding the common -PPGFSPF- and -PPGFTPF- internal amino acid sequences of ranid frog skin BRPs and a UPM primer (supplied with the kit). The 3′-RACE reactions were purified and cloned into pGEM^®^-T Easy Vector (Promega Corporation, Southampton, UK) and sequenced using an ABI 3100 automated capillary sequencer. The 5′-RACE PCR employed an antisense primer (AS: 5′-CCGTATGTCATTTAGCTAAATGATGA-3′), which was designed to a highly conserved domain located within the 3′ non-translated region of the obtained 3′-RACE transcripts, and a UPM primer. In addition, the 5′-RACE products were gel-purified, cloned, and sequenced.

### 4.3. Identification and Structure Characterization of a Novel BRP from the Odorrana livida Skin Secretion

An aliquoted sample of five milligrams lyophilized *Odorrana livida* skin secretion was dissolved in 1 mL trifluoroacetic acid (TFA)/water (0.05/99.95, *v*/*v*), followed by centrifugation to clarify the sample solution. The clear supernatant was decanted and pumped into an analytical reverse phase HPLC column (pheomenex C-18, 250 × 10 mm) for fractionation by using a Cecil Adept 4200 HPLC system (Amersham Biosciences, Buckinghamshire, UK). The linear gradient elute method was used from TFA/water (0.05/99.95, *v*/*v*) to TFA/water/Acetonitrile (0.05/19.95/80.00, *v*/*v*/*v*) over 240 min at a flow rate of 1 mL/min. The fractions were collected automatically at one-minute intervals; the effluent absorbance was continuously monitored at 214 nm. Fractions were lyophilized and stored at −20 °C prior to being subjected to the smooth muscle assay. Meanwhile, the molecular masses of polypeptides in the collected fractions were analysed through matrix-assisted, laser desorption/ionization, time-of-flight mass spectrometry (MALDI-TOF MS) on a linear time-of-flight Voyager DE mass spectrometer (PerSeptive Biosystems, Framingham, MA, USA). The sample was analysed in positive detection mode using α-cyano-4-hydroxycinnamic acid as the matrix. Internal instrument calibration was performed by using standard peptides of established molecular masses, which provides the determined accuracy of ±0.1%. Fractions with the masses coincidence with those deduced mature peptides from the molecular cloning were subjected to MS/MS fragmentation sequencing analysis by using a Thermoquest gradient reversed phase HPLC system (Thermo Fisher Scientific, San Francisco, CA, USA), fitted with an analytical column (C-18), and interfaced with a Thermoquest LCQ™ Deca electrospray ion-trap mass spectrometer (Thermo Fisher Scientific, San Francisco, CA, USA).

### 4.4. Chemical Synthesis of Novel BRP (RAP-L1, T6-BK) and Its Modified Analogue RAP-L1, T6, L8-BK

Once the unequivocal primary structure of the novel bradykinin-related peptide had been determined through MS/MS fragmentation sequencing and molecular cloning of its biosynthetic precursor-encoding cDNA, Fmoc chemistry based solid phase peptide synthesis was performed to produced RAP-L1, T6-BK peptide replicates by using Tribute^®^ Peptide Synthesizer (Protein Technologies, Inc., Tucson, AZ, USA). The analogue RAP-L1, T6, L8-BK was also synthesized with the same method. All synthetic peptides were purified with reverse phase HPLC. The authenticity of their primary structures and purity were confirmed via MALDI-TOF MS and LCQ MS/MS fragmentation sequencing.

### 4.5. Preliminary Study of Inherent Cytotoxicity of the Novel BRPs

Prior to the smooth muscle assay for evaluating the pharmacological actions of BRPs, the MTT cell viability assay was performed. Cultured human microvessel endothelial cells (HMECs) were used to assess the possible influence of inherent cytotoxicity caused by the synthetic novel BRPs. In detail, the HMECs were seeded in 96-well plates with the density 5000 cells/well. After overnight incubation, HMECs were subjected to 6 h starvation prior to being treated with synthetic BRPs (RAP-L1, T6-BK and RAP-L1, T6, L8-BK) ranging from 10^−11^ M to 10^−5^ M for 24 h. Cell viabilities were assessed by MTT assay, as described previously [28].

### 4.6. Ex Vivo Rat Bladder, Ileum, Uterus and Tail Artery Smooth Muscle Pharmacology

Wistar rats (200–250 g) were euthanized by asphyxiation with carbon dioxide in accordance with institutional animal experimentation ethics and UK animal research guidelines. The rats were placed on their dorsal surfaces, an incision was made along the mid ventral line towards the bottom of the abdomen and subcutaneous fat was carefully removed. The smooth muscle tissues of bladder, ileum, uterus, and tail artery were dissected gently followed by being placed in ice-cold Krebs’ solution immediately (118 mM NaCl, 4.7 mM KCl, 25 mM NaHCO_3_, 1.15 mM NaH_2_PO_4_, 2.5 mM CaCl_2_, 1.1 mM MgCl_2_ and 5.6 mM glucose), and equilibrated with a mixed gas (95% CO_2_ and 5% O_2_). The tissues were dissected and mounted as shown in the table to obtain maximum viability and to achieve the best response (Table 1).

After dissection, small strips of particular tissues were connected to a triangular hook. Then, the removed tissues were mounted to the force transducers in a 2 mL organ bath which were perfused with Krebs’ solution (118 mM NaCl, 4.7 mM KCl, 25 mM NaHCO_3_, 1.15 mM NaH_2_PO_4_, 2.5 mM CaCl_2_, 1.1 mM MgCl_2_ and 5.6 mM glucose) at 37 °C and constantly bubbling 95% O_2_ and 5% CO_2_ gas mixture. The prepared tissues were attached to the force transducer in order to detect the tension changes. The responses of tension change were recorded and amplified through pressure transducers connected to a PowerLab System (AD Instruments Pty Ltd., Oxford, UK). The peptide solutions of RAP-L1, T6-BK and RAP-L1, T6, L8-BK were prepared in the range of 10^−11^ M to 10^−5^ M with fresh Krebs’ solutions to construct the dose–response curves. Each concentration of individual peptide was applied to a minimum of six muscle strips.

For the rat tail artery preparations, endothelial linings were essentially removed from the dissected smooth muscle, a basal tension of 0.5 g was applied to the organ bath incubated tail artery before adding phenylephrine (1 × 10^−5^ M) for 10 min to achieve constriction plateaux. To further exclude the possible vasorelaxant effect caused by endothelial-derived nitric oxide (NO), tail artery smooth muscles were pre-treated with/without 0.5 µM eNOS (endothelium nitric oxide synthase) inhibitor *N*_5_-(1-Iminoethyl)-l-ornithine dihydrochloride (L-NIO, Tocris Biosciences, San Diego, CA, USA) for 20 min, prior to performing a BK sequential concentration (10^−11^ M to 10^−5^ M) -induced vasodilation.

For urinary bladder and ileum smooth muscle preparations, the bladder and ileum strips were gradually exposed to increasing tension until 0.5 g was reached and maintained. The preparations were then exposed to peptides as described above and relative changes in tension were recorded.

For uterus smooth muscle preparations, female Wistar rats were euthanized by carbon dioxide. The dissected uterine horns were mounted in each organ bath, incubated with 37 °C Krebs’ solution for 10 min with no tension. The uterus strips were gradually exposed to increasing tension until 0.5 g was reached and maintained. The uterus smooth muscle preparations were exposed to peptides as described previously and, changes in spontaneous contraction frequencies, instead of tension change, were recorded.

To examine what type of BK receptors in rat tail artery smooth muscle were targeted by BRPs, a single 10^−6^ M dose of BK B1 receptor antagonist R715 and/or the BK B2 receptor antagonist, HOE140 (Sigma Aldrich, Irvine, UK) were applied to artery smooth muscle preparations prior to peptide treatment.

Data from this study were analysed by one-way ANOVA with Bonferroni’s post-test and two-way ANOVA using GraphPad Prism software (version 5.01, San Diego, CA, USA). A *p* value less than 0.05 was considered significant.

## Figures and Tables

**Table 1:** The cutting size, mounting way and basal tension of rat smooth muscle tissues.

Smooth Muscle Tissues	Cutting Size	Mounting Way	Basal Tension (g)
Bladder	10 mm × 2 mm (length × width)	Hooked longitudinally	0.5–1.0
Uterus	Entire Uterine horns	Mounted horizontally	
Ileum	0.5 cm length ring	Mounted horizontally	
Proximal rat tail artery	2 mm width ring	Mounted horizontally	

## Supplementary Materials

The following are available online at www.mdpi.com/2072-6651/8/10/283/s1, Figure S1: Dose–response curves of administration of BK and BRPs using rat arterial smooth muscle. Figure S2: MALDI-TOF (Perceptive Biosystem, Bedford, MA, USA) mass spectrum of synthetic peptide RAP-L1, T6, L8-BK.

## Data Deposition Footnote

The nucleotide sequence of the cDNA encoding the novel bradykinin-related peptide RAP-L1, T6-BK precursor from the skin secretion of *Odorrana livida*, has been deposited in the EMBL Nucleotide Sequence Database under the accession code: LT576450.
