# Supplementary material for: Ex Vivo Smooth Muscle Pharmacological Effects of a Novel Bradykinin-Related Peptide, and Its Analogue, from Chinese Large Odorous Frog, Odorrana livida Skin Secretions
Source: Toxins (Basel). 2016 Sep 27;8(10):283. doi: 10.3390/toxins8100283 (PMC5086643; doi:10.3390/toxins8100283)
Supplement: Supplementary file 1 [file toxins-08-00283-s001.pdf]

Supplementary Materials: Ex vivo Smooth Muscle Pharmacological Effects of a Novel Bradykinin-Related Peptide, and Its Analogue, from Chinese Large Odorous Frog, *Odorrana Livida* Skin Secretions

Jie Xiang, Hui Wang, Chengbang Ma, Mei Zhou, Yuxin Wu, Lei Wang, Shaodong Guo, Tianbao Chen and Chris Shaw

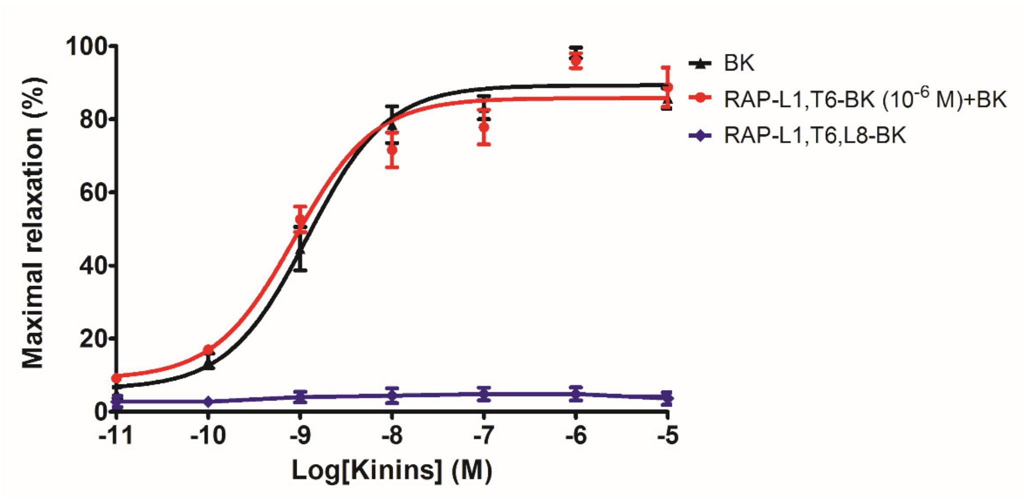

Figure S1. Dose–response curves of administration of BK and BRPs using rat arterial smooth muscle.

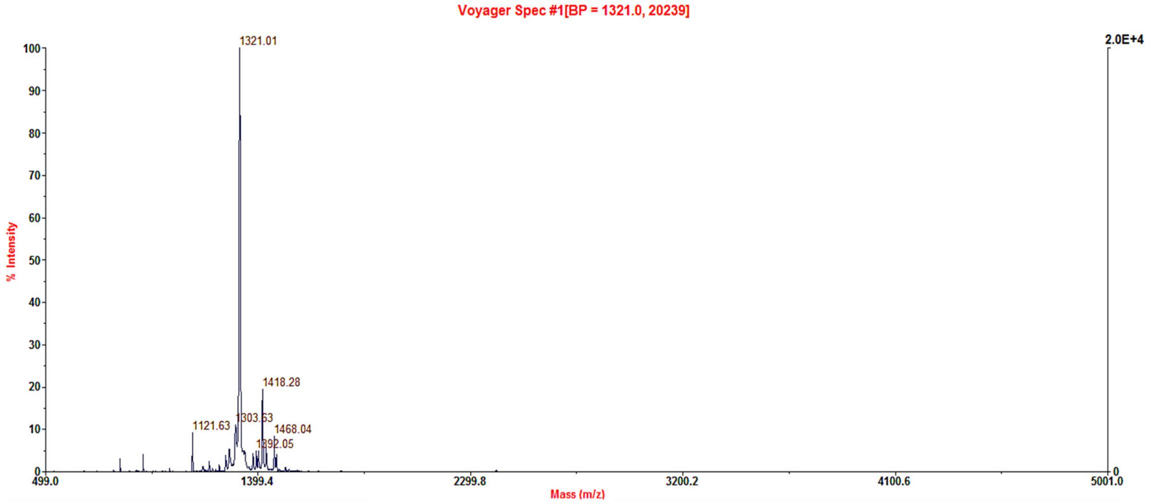

Figure S2. MALDI-TOF (Perceptive Biosystem, Bedford, MA, USA) mass spectrum of synthetic peptide RAP-L1, T6, L8-BK.
